# Blistering Plaster Prepared with Chloroform

**Published:** 1855-11

**Authors:** 


Blistering Plaster Prepared with Chloroform.—Dr. Landerer, of Athens, recommends, as an improved mode of making blistering plaster, the preliminary digestion of the powdered cantharides for several days, at a gentle heat, in a sufficiency of chloroform to moisten it. The mass is then to be added to the plaster half cold, taking care to avoid inhaling the chloroform which becomes volatilized.—(*Gaz. des Hôp.* No. 69.) *Ibid.*

